# The Chicago Medical Society

**Published:** 1887-07

**Authors:** 


### THE CHICAGO MEDICAL SOCIETY.

### STATED MEETING, JUNE 20, 1887.

The President, W. T. Belfield, M.D., in the chair.

Dr. Harold N. Moyer and Dr. Alfred Hinde presented a paper on

"PERIODICALLY RECURRING OCULO-MOTOR PARALYSIS,"

with the report of a case.

The patient had suffered from her seventh year with complete paralysis of the third nerve in all its branches. The attacks recurred at varying intervals of from four to six months and were always accompanied by headache and vomiting. The attacks have grown more frequent of late; and, since the earlier ones, recovery from the paralysis has not been complete.

The literature of the affection was given, and mention made of some sixteen cases so far reported. The etiology, pathology and prognosis also received consideration.

DISCUSSION.

Dr. W. F. Coleman : This paper is so excellent and unique, I certainly have nothing like it in my own experience to relate. I have perhaps fifteen or twenty text-books in English, German and American, but none of them refer to periodical paralysis of the motor oculi nerve, and I have seen no reference to it in any work on the eye. Isolated paralysis of the whole third nerve, even of organic origin, is a rare affection without involving other cerebral nerves. As the interesting point in the paper is the source and pathology of the disease, I will not undertake to discuss any other point. These cases are more interesting, if possible, to the general practitioner than to the oculist, on account of the connection between cerebro-spinal disease and paralysis of ocular muscles. The post-mortem in three cases showed that the nerve trunk itself was involved, which would point to an organic origin of those cases of periodic motor oculi paralysis, and I suppose in those cases relapses occurred from inflammatory exacerbations. I agree with Dr. Moyer that a large majority of the cases related would in all probability be functional. In his own case there were symptoms of migrain, viz., headache and vomiting, accompanying the motor oculi paralysis. Organic lesions of the brain, such as basilar meningitis, etc., are in the great majority of cases, say 75 per cent., accompanied by optic neuritis. Since in the sixteen cases related by Dr. Moyer there is no mention, I believe, of optic neuritis, I infer the motor oculi paralysis was not due to organic disease of the brain, but to functional causes.

Dr. C. F. Sinclair : It has never been my experience to have met with a case precisely similar to that to which Dr. Moyer has referred, neither have I met with one in my reading. But it seems to me from the number of cases to which he refers, all with symptoms resembling one another in one or more particulars, and in all of which was found this element of periodicity, that we have almost data for the establishment of a new and definite pathological condition which would lead the way to very broad investigation in ophthalmic science. The paper is particularly interesting to me because one case to which reference has been made resembles in a very great degree one which came under my observation some weeks ago. It was a case of paresis of only one of the branches of the oculi motor nerve, but it was sufficiently marked to cause a high degree of ptosis. The element of periodicity was present in this case, and that, I take it, is the peculiar feature in the cases cited by Dr. Moyer. In the case to which I refer were associated with the paresis other peculiar and morbid conditions. My patient was a young woman, 17 years of age, of German parentage. When I first saw her I noticed at once a peculiar expression of the eyes, which I found came from the fact that one was a light, almost sky blue, and the other gray. She told me that three years previous both of her eyes had been dark brown and that two years ago the left eye began to grow lighter in color, changing to a light gray. That is a phenomenon which I must confess never to have heard of before, where the pigment in the eye of a person of that age has undergone such marked changes within a period of three years without any evidence of iritic inflammation to account for them. Dr. Moyer, I believe, mentions a case where this oculi motor paralysis had occurred in the spring successively on several different occasions. The attacks which my patient suffered occurred also in the spring, coming on each time in the same month and nearly the same day. When she came under my care she was suffering the third attack, which was precisely similar in every respect to the two preceding. There were several days of extreme languor and drowsiness, during which time there was an irresistable impulse to cry on the slightest occasion. Then followed the ptosis, accompanied with severe neuralgiac pain through the temple and over the vertex on one side, and at the same time a small infiltrated ulcer made its appearance on the lower margin of the cornea. The usual local means I found entirely unavailing in checking the attack. This case suggested to my mind the possibility, and the suggestion has been renewed with added force to-night, that as ophthalmic surgeons we are too apt to dwell solely upon the local manifestations of disease in the arteries, tunics of the eye and its appendages, and to limit our treatment entirely to local treatment. It seems to me that it is time for us to recognize the fact that in many of the morbid conditions of the eye which we have been accustomed to consider as altogether of local origin, the causation lies in reality much deeper, either in the great nerve centers or in diseased conditions of other and perhaps remote organs, and unless we direct our remedial measures to these our results can only be transient and unsatisfactory.

Dr. Moyer, in closing the discussion, said: Here, as in similar cases, we need to have an exact definition of what we mean by the term "functional." If we call only those lesions organic in which there is no absolute degeneration or destruction of the nerve cells, and speak of transitory congestion as "functional," then we must all admit that many, perhaps most, of these cases fall within the latter class. But the term used in its strictest sense—that is, in the sense that hysterical or reflex conditions are functional—then the only case so far as our observation extends, ever reported is the one described by Jacobi. Every case of the seventeen so far reported, including our own, has been progressive. The recovery earlier in the disease was complete after each attack. Later a certain amount of paralysis would remain. Mobius refers the paralysis to an inflammation or congestion of the nucleus of the nerve, coming on periodically, and which in time results in a certain amount or even total paralys's. The anatomical diagnosis is the most interesting feature of the case. We have purposely refrained from offering any theories of our own regarding the probable seat or nature of the difficulty; yet we confess to a strong leaning toward the theory of a nuclear degenerative process. As Dr. Coleman has pointedly said, this disease in not associated with any changes in the retina or optic disc, which rather militates against the theory of a basilar meningitis as the basic pathological condition. We must however admit that the two earliest cases that came to a post-mortem examination were basilar in their nature, and in the third an enchondroma had impaired the integrity of the nerve.

Report:

"CANCER OF THE TESTICLE, WITH SECONDARY PLEURISY,"

with exhibition of pathological specimens, by Dr. John A. Robison.

The subject from which this specimen was obtained was a Frenchman, aged about 24 years, who was admitted to the Cook County Hospital May 1st, for a tumor of the left testicle, pronounced sarcoma, of eleven months standing. An operation was advised and refused, the patient leaving the hospital. A few days ago he returned with all the signs and symptoms of effusion in the left pleural cavity. The symptoms were so severe that I ordered him to be aspirated, and remarked that the product of aspiration would undoubtedly be bloody serum. So it was. The patient lived only a few hours, and the autopsy demonstrated that there was a large cancerous tumor, six inches in diameter, in the scrotum involving the left testicle; also, a tumor nearly as large in the abdominal cavity, with secondary cancerous deposits over the pleuræ of both lungs at the apices

The symptoms of cancer of the pleura are always obscure, unless we have the history of primary cancer in some other part of the body. This is the fourth case of pleural cancer I have seen, the cause in these cases being respectively cancer of the kidney, as diagnosticated by Dr. Fenger, cancer of the humerus, cancer of the liver, and the present case of cancer of the testicle.

The exudation in cancerous pleurisy is serum mixed with blood and tissue detritus, if the cancer granulations are degenerating. I believe the bloody serum is almost pathognomonic of cancer, as about the only diseases in which we obtain such a fluid on aspiration is in pernicious, or malarial, anæmia, phthisis and purpura, and even in these diseases the exudation is more likely to be purulent.

Two causes for the hæmorrhagic effusion have been assigned:

1. The effusion is the result of the breaking down of the cancerous granulations.

2. It is the result of the cancerous cachexia, where the blood is impoverished, the absorbent vessels weak and osmosis very rapid.

I am inclined to believe the effusion is due to inflammation produced by the presence of these foreign bodies—the graulations—and the breaking down of these granulations.

Remarks: These cases teach me this lesson: that oftentimes patients who suffer from cancer of some portion of the body amenable to treatment, frequently die of secondary cancer of the pleua, which might have been averted had the primarily diseased tissue been entirely removed. This is certainly true of many cases of cancer of the genital organs, breast, bones, etc.

The specimens were then exhibited. They consisted of the original cancerous tumor of the testicle, the collapsed left lung, whose pleural surface was thickly covered by the reddish exudate, and the right lung, in the apex of which was a cancerous growth one and a half inches in diameter.

DISCUSSION.

Dr. H. A. Johnson: Cancer of the lungs occurs primarily but very rarely. I have seen two cases. Recently I saw a case which I supposed to be cancer of the lung in which there was primary cancer of the larynx. It was interesting as the only case I have seen where cancer of the larynx has been followed by secondary cancer.

Dr. Robert H. Babcock: I am particularly interested in the case just mentioned, especially in that part pertaining to cancer of the pleura. I was reminded of a case that it was my good fortune to see in Munich, by the remark that serum mixed with blood is almost pathognomonic of cancer of the pleura. In the hospital in charge of Von Ziemssen was a man who had been in several hospitals, and in whom Bamberger, in Vienna, had diagnosticated cancer with pleurisy of the left side. The same diagnosis was made by Von Ziemssen and his assistant. I examined the case, and although I do not remember all the signs present, I remember being struck with the extreme flatness over all the left side, the absence of respiratory sounds, the resistance of the side. The assistant informed me he had been tapped and a bloody discharge obtained, which seemed to confirm the diagnosis. A day or two previous to my leaving Munich I inquired about this case. The man had died, and they found no cancer whatever, but a remarkable thickened condition of the pleuræ; the fibrinous deposit having become very highly organized. It was the oozing from the blood vessels which were severed in the tapping which had given the bloody discharge, and misled such celebrated men as Bamberger and Von Ziemssen.

Dr. Elbert Wing: I do not know of a case that is similar to this, but I remember a case which I saw diagnosticated by the late Prof. Frerichs, of sarcoma of the lung, primary. The symptoms were those of severe, acute lancinating pain, and rapid development of the tumor, with no family history showing what the lesion might be. The physical signs were those of consolidation at the right apex. Everything else being excluded in the diagnosis, Prof. Frerichs said it was either a carcinoma or a sarcoma, and that as sarcoma of the lung was more common he presumed it was sarcoma. After a post-mortem microscopical examination Prof. Frerichs' assistant said it was a case of sarcoma. Prof. Virchow's assistant said that it was carcinoma. It was probably one of those cases that are right on the border line between the two tumors, viz., sarcoma-carcinomatodes, and the examination was not complete. In regard to the bloody effusion, or fluid, in the pleural cavity, I happened to hear Prof Frerichs say he always regarded that as an indication of tubercular pleuritis.

Dr. Frank Billings: The only thing of interest I have to offer is in regard to the color of the serum. Profs. Nothnaegel, Bamberger, and Kundrat, of Vienna, teach that bloody serum in *any serous* sac is due to (1) a malignant growth (carcinoma, sarcoma, etc.), (2) tuberculosis, and (3) to poisons that cause fatty degeneration of the walls of blood vessels and consequent capiliary hæmorrhage (phosphorus). The presence of the bacillus tuberculosis would easily differentiate the first two causes, if the growth in the lung *were* primary carcinoma. The last cause would hardly be mistaken for the first two.

Dr. J. J. M. Angear: I do not know, Mr. President, that I have anything further to add to this subject, except as far as my observation goes, cancer of the pleura has alwas been secondary. I have seen cases of secondary cancer, not only of the pleura, but the sub-serous tissue of the pleura, the connective tissue around the base of the heart infiltrated with malignant growth, as well as the substance of the lungs. Bloody serum comes from extreme passive congestion—impoverished condition of the blood, as well as from the breaking down of the cancerous growth. Our knowledge of the primary cancer aids us greatly in recognizing the cachexia. Taking these two things together with a bloody serum in the pleura, the diagnosis of secondary cancer of the pleura becomes simple.

Dr. John A. Robison, in closing the discussion, said: The diagnosis of cancer was simply suggested to me by the nature of the tumor, the slow growth, and the amount of pain the patient had, etc., and finally the character of the effusion. The question that puzzles me most is, whether, when aspirating a patient we get this reddish fluid, it is not a pathognomonic sign of cancer. It has been my good fortune to have had an extended experience in the aspiration of a large number of patients, tubercular and otherwise, and in cases where I obtained well-marked physical signs of tuberculosis (it was before we examined for bacilli) never have I obtained this reddish fluid, and in all cases of cancer which I have aspirated I have obtained this reddish fluid. It was the same color each time. I am groping about for an opinion, but would not say positively that when we get that kind of fluid the patient has cancer.

Dr. Robert H. Babcock reported

"A CASE OF PERICARDITIS AND ENDOCARDITIS."

Mrs. B., aged 23, Norwegian, consulted me April 30, 1887, complaining of præcordial pain and dyspnoea. History was as follows: Her mother died of heart disease; father in good health; youngest sister has heart disease as result of rheumatism; strong family predisposition to inflammatory rheumatism. After birth of only child, six years ago, patient was ill a year of rheumatic fever but had no knowledge of heart or lung complications at that time. Since fore part of last winter has been suffering with rheumatism in ankles, left knee and corresponding shoulder. For past three weeks has been unable to lie down because of pain in the chest and shortness of breath. No history of any lung trouble.

Her symptoms were acute præcordial pain, intensified by dorsal decubitus, pressure and full-inspiration; also slight joint pains. Cough troublesome, with mucus expectoration; sleep very broken; appetite poor; constipation; dry, coated tongue; no fever; pulse 120, feeble and unequal. Respiration short, hurried and sighing.

Examination of heart revealed impulse in fifth interspace outside of mammary line, feeble and preceded by a short thrill, a very marked pulsation in the third left interspace close to sternum, due to closure of pulmonary valves; considerable increase in all diameters of the area of praecordial dullness, particularly above and toward the left, where the lung border was retracted and fixed. Upon auscultation was heard a short, rough, presystolic murmur at the apex followed by a higher pitched systolic whiff, which latter murmur was propagated into the axillary region and on to the base of the heart. Over the body of the organ was also heard a rough sound of rolling characters, not unlike the rhythm produced by the hoofs of a galloping horse. Owing to the weakness and evident misery of the patient the lungs were not examined, a fact I now greatly regret. The diagnosis was acute simple endocarditis, supervening upon chronic endocarditis changes; mitral stenosis and insufficiency; pericarditis and possibly myocarditis; chronic inflammatory rheumatism. Rest in bed, hot applications to the praecordial, salicylate of sodium and digitalis in small doses were prescribed. In a day or two the digitalis was discontinued, however.

Under this treatment the pain in the chest was abated and the patient's condition improved, although the pains in the joints continued. At the end of ten days, upon her urgent solicitation, she was permitted to get up and lie on the lounge. The pulse never became slower than 110 to the minute, which I considered a bad indication from the close of the first week. At the end of another ten days or so she took to her bed complaining of anorexia nausea, and inability to retain food. The tongue was heavily coated and dry, and slight catarrhal icterus developed. A few small doses of calomel, followed by an alkali and simple bitter, afforded prompt relief, and for the next week the patient appeared to improve in some respects. Her condition was tolerably comfortable by day, but by night she suffered much from restlessness, pain and dyspnœa. Repeated examination of the heart disclosed no increase of praecordial dullness, while the endocardiac and friction murmurs became even more intense and characteristic, except for a short time, during which the urine was very scanty. The lessened intensity was probably due to effusion, as very likely was also the abatement of pain. Under the effect of a diuretic the friction sounds reappeared with added distinctness. June 2nd she suddenly developed an acute attack of rheumatism of the left wrist and thumb joints, with coincident aggravation of chest symptoms. A double two and front-friction sound became remarkably distinct over and about all the ensifom cartilage. Temperature was 102 F. Salicylate of soda brought prompt relief from pain, with, however, but slight effect on the temperature. The urine, which was intensely acid, was rendered alkaline by bicarbonate of potash.

Sunday, at 2 o'clock, A.M., she was seized with a sharp pain in the chest and epigastrium, lasting two hours, only relieved by opium. When I visited her next day she was suffering much from dyspnœa and exquisite pain in the epigastrium. The lightest pressure could not be borne, while the abdominal walls were very tense. Satisfactory percussion and palpation were out of the question, and hence I was greatly puzzled to account for her symptoms. Her pulse was 138 and very feeble. Cursory examination of the lungs disclosed mucous rales at right base posteriorly, and slight dullness below the angle of left scapula, with tympanitic note above. A loud, rough systolic murmur obscured the respiratory sounds, but the voice was heard with great distinctness. Hot applications to the epigastrium afforded relief to pain and the abdomen became less tense. At 11 P. M. of Wednesday there was sudden effervescence followed by profuse perspiration for the balance of the night. Thursday P. M. she was without fever and comparatively free from pain. Upon examining the chest I found the physical signs very much as three days before, except that there were moist, for the most part subcrepitant, rales over the area of dullness; voice very distinct. Cough was well-nigh impossible by reason of pain; the scanty expectoration had a slight brownish-red tinge. I made up my mind that I had a pneumonia to deal with which had sneaked in and escaped my notice. Further reflection, however, convinced me that this opinion was probably an error, and that there was a hydrostatic congestion with bronchitis.

The patient's strength steadily waned, and the following evening death closed her struggles. She and I were both handicapped throughout, as she had no good nursing, but was dependent upon the tender (?) mercies of indifferent neighbors. So far as possible, nevertheless, the treatment was supporting and anti-rheumatic, although in combating the rheumatism with potassium salts and salycilate of soda I had to exercise caution for fear of depressing the already weakened heart. Death took place from exhaustion

A few more words and I shall have finished. The chief symptom complained of was in the præcordia and between the shoulders behind This was probably due directly to the acute pericarditis which the autopsy showed had been limited to a small area on the anterior service of the left ventricle just below the origin of the aorta, and to the posterior aspect of the left auricle, which latter situation, it seems to me, would account for the interscapular pain. Since the necropsy revealed no cause for the epigastric pain and tenderness, it is reasonable to assume that these were reflected. I have since found a statement in Zeimssen's Cyclopædia to the effect that this epigastric pain and tenderness on external pressure is not an uncommon occurrence in pericarditis.

I of course suspected the existence of pericardial effusion, but did not at any time demonstrate its existence to my satisfaction, because, owing to the denudation of the heart already present, it did not influence the area of cardiac dullness and did not abolish the friction sounds, as I expected an effusion would do. I have since learned that, according to Ceuka, as stated by Bauer in Ziemssen's Cyclopædia, friction sounds have been audible even when the pericardium held a quart of fluid.

Within the left pleural cavity were, as was estimated, eight or ten ounces of serous fluid which I had not diagnosticated. Under favorable conditions this small quantity of liquid ought to be discovered, but in this case the conditions were complex and so modified physical signs as to mislead me. Had the layer of fluid been of sufficient depth to intercept the sound waves, on their passage through the pleural cavity, the complete or partial suppression of the respiratory and vocal sounds would have revealed the presence of the effusion. In reality, however, the hypostatic congestion, discovered post-mortem together with the comparative condensation of the lungs, occasioned by the enlarged heart and effusion, produced a condition calculated to intensify the transmission of sound waves instead of deadening them. Hence, the distinctness with which I heard the rales, the voice and breath sounds, although, owing to the loud, harsh systolic murmur, I could not determine the quality of the respiration. The very intensity with which the mitral bruit was transmitted still further served to mislead me. Yet, it may be asked if the effusion was so slight, how could it give rise to a tympanitic percussion note above the level of the fluid ? In fact, this sign in conjunction with the consolidation below, as I supposed, was very puzzling to me at the time. Reflection, however, enables me to explain it, I think. Tympany above the level of an effusion is explained by Gerhardt as being due to the relaxation of the lungs caused by the pressure of the fluid. In other words, the normal tension of the lung is lost. In this case, whereas the effusion alone may not have sufficed to occasion material relaxation of the lung, it was assisted to this end by the pressure of the heart in front, which, as shown by the autopsy, had crowded aside the anterior border of the lung where it had become fixed in its new position. Thus the normal lung tension was overcome.

Whether my failure to recognize the pleuritic effusion intra vitam can be excused on the ground of the complexity of the conditions present or not, it has proved a most salutary lesson to me; and it was in the hope that others might perchance profit by it that I have ventured to trespass so long upon your time and attention.

Post-mortem notes: Family predjudice prevented a thorough examination.

Heart.—There was about four ounces of clear, reddish serous fluid in the cavity of the pericardium (quantity estimated). The parietal layer of the pericardium was throughout its extent very greatly thickened and covered with projections and folds of fibrinous deposit, disposed in somewhat parallel ridges, running irregularly in direction, and most markedly present over the anterior and posterior surfaces of the sac; these areas corresponding in situation to similar ones in the visceral layer of the heart. Their appearance was partly villous and partly velvety. The visceral layer of the pericardium was also involved throughout its entire extent. The fibrinous deposit was thickest over the anterior surface of the heart, where it was thrown into folds and ridges whose general direction was at right angles to a line passing from base to apex of the heart. Many of these ridges were from two to three sixteenths of an inch in heighth, and they were most abundant and largest about the middle of the anterior surface of the heart.

Over the posterior surface of the heart the fibrinous layer is, upon the whole, much thinner, but the projections are more distinctly villous, many being, in fact, severed bands of adhesion, and the general appearance somewhat reticulated.

On the anterior surface of the heart, immediately below the line of the aortic valves, there was a recent circular adhesion, one inch and a quarter in diameter, soft and easily separated.

Over the posterior surface of the left auricle the inflammatory process was evidently quite recent. Immediately above this spot the pleural surfaces were involved, and there was a small area just there of pleural adhesion.

The position of the heart was such that the right border lay transversely and the apex was three inches to the left of the median line. The heart had completely displaced that part of the lung which lies in front of the heart. The auricles of the heart were filled with dark firm clots, and the ventricles were approximately empty.

The endocardium of the left ventricle was of a dull, turbid, grayish color, mottled with occasional small, yellowish-gray points, but smooth and glistening.

There were vegetations upon the aortic valves disposed in a highly characteristic manner. Just below each corpus aurantii there were knots or clumps of vegetation about the eighth of an inch in diameter and height, somewhat pyramidal in form, and extending in a crescentic line from these points; along the so-called line of contact of the valves was a clearly marked row of smaller vegetations. The vegetations were most abundant on those segments of the valves which are opposite the point of origin of the coronary arteries. There were a few very small patches of fibrous tissue at the base of the aorta.

The anterior segment of the mitral valve was markedly thickened throughout its extent, and it was also moderately contracted. The posterior segment was similarly thickened and so adherent to the contiguous ventricular wall that only about one-fourth of an inch of its lower border was free. The edges of both segments were about one-eighth inch thick and studded with vegetations which had a distinctly granular appearance and feeling. The result of the contraction of the segments was a moderate stenosis of the orifice. The left auricle was greatly dilated and its endocardium thickened and studded with small irregelarly-shaped fibrous patches. Its color was a turbid, yellowish gray.

The muscular tissue of the heart was dull, pale and flaccid. The thickness of the wall of the left ventricle (muscular tissue alone) was one-half inch. The heart measured 5¼ by 5 inches, and weighed 1 pound 2 ounces (avoirdupois). There was a moderate dilatation of the ventricles, but the enlargement of the organ was more of the nature of an hypertrophy than a dilatation. Except as mentioned, the changes in the heart were not of special pathological interest.

Lungs.—There is a fluid in the left pleural cavity reaching, as the body lies, in the dorsal decubitus to the anterior axillary line. This fluid was not measured but was approximately eight to ten ounces, clear and slightly reddish. The lower border of the upper lobe of the left lung corresponded anteriorly to the third rib and was adherent. This line of adhesion, as well as a few others over the surface of the lung, was not of very recent origin, but yet was easily broken down. That part of the pleural surfaces, however, immediately next the base of the heart was very firmly adherent. In this lower part of the left lung there was a moderate degree and amount of hypostatic hyperæmia and œdema. There were a few old and moderately firm adhesions over the right lung. The spleen was of less than normal size and soft and degenerated. The liver was rather pale but in a state of moderate passive hyperæmia. There were a few very thin and not very recent bands of adhesion between its left lobe and the diaphragm. The other organs were not examined.

The diagnosis, limited to the organs examined, was: chronic pericarditis, serofibrosa, and endocarditis with recent acute exacerbations of both with effusion, chronic myocarditis, and chronic and acute circumscribed pleuritis of the left side with effusion and pulmonary hyperaemia and œdema; chronic pleuritis of the right side; circumscribed peritonitis (diaphragmatic), subacute splenitis, passive hyperæmia of the liver.

Dr. John A. Robison: I think Dr. Babcock was perfectly excusable in not detecting the pleurisy, because often times when there is not more than eight or ten ounces in the pleural cavity and no complicating disease, if the respiratory sounds are very intense, or if the lung has not been pressed upon to any great extent, there will be still a sufficient amount of resonance on that side to overcome any dullness the small amount of effusion will cause. I know in a great many cases it is a very puzzling question to a person making an examination to tell whether there is any fluid in the cavity or not by the physical signs.

---

### STATED MEETING, TUESDAY, JULY 5.

---

The President, W. T. Belfield, M.D., in the chair.

Dr. E. B. Weston reported

"CASES OF SYNOVITIS OF THE KNEE JOINT."

Synovitis, though a surgical disease, is one which the general practitioner often treats—is often compelled to if he have a country practice —and there is no reason why he should not treat it well and successfully.

A slight synovitis may recover rapidly without treatment, and so these cases get well when treated with a poultice, or painted with tincture of iodine. But the physician who makes this his routine practice will not have many "flattering successes," and the number of cripples in his neighborhood may be monuments to his want of thoughtful attention.

I have four cases of synovitis of the knee to report. Not that they present points of peculiar nterest, but to emphasize their importance, even though I disclose my own mistakes in doing so.

Case I.—A. B., Irish, aged about 50, laborer. I met him on the street Jan. 1, 1885, when he told me he had pain in his left knee; that he did not know of injuring it in any way. Having a physician's dislike for using "all out doors" for his office, and being consulted wherever he chances to meet a would be patient, I gave him little attention, and advice of a very general nature. One week later he sent for me. I found him in bed suffering severe pain, knee distended with fluid in joint cavity, leg flexed. He refused to have fluid evacuated, or even to have leg put on splint, although I plainly told him of the probable consequences of not doing so. Then, instead of leaving the case, I allowed him to be his own physician, while I continued to make daily visits and suggestions.

I have no full notes of the case, but it went from bad to worse. Abscesses formed in the thigh, which were opened as they developed, drainage tubes passed in various directions, the limb becoming honey-combed by the burrowing pus. These cavities were frequently syringed with carbolized water and the limb kept on a pillow of oakum. He was given a nourishing diet, stimulants and tonics. But he gradually lost flesh and strength, temperature increased, and on the 1st of March he died of sepsis and exhaustion.

Case II.—C. D., American, aged about 45, by profession a lawyer. First saw him March 4, 1886, when he told me his left knee had pained him quite constantly, but not very severely, for about ten days. He thought he had taken cold in it while sitting at his desk, under which he had felt a draught. I found knee moderately enlarged, joint distended with fluid and hot and painful. I ordered him to bed and had applied an anodyne liniment and warm fomentation.

This treatment was continued for three day without benefit, when I opened the synovial cavity with a bistoury and evacuated about eight ounces of light straw colored, probably nearly normal, synovial fluid. He was at once relieved of pain, the limb put on a well padded posterior splint, and an elastic bandage applied. The fluid gradually reaccumulated, and in four days the joint was again full, hot and painful. I now aspirated it and reapplied bandage. There was again a slight filling, but not sufficient to necessitate opening. The patient steadily improved, and in a few weeks had perfect use of the joint.

Case III.—E. F., a Swede, aged 25, painter. Feb. 19, 1886, at noon, while on his way to work he fell, striking his right knee on the ice. It caused him severe pain for a short time, but he worked the whole afternoon, standing on a step-ladder, without much discomfort. At night the knee began to swell and pain him intensely When I saw him the knee was hot, very painful and moderately distended, and leg flexed. A liniment composed of opium, aconite and menthol was applied, with warm fomentation, and opium given internally. No improvement taking place, and the joint becoming much distended, on the 22nd instant I opened it with a bistoury, letting out a large quantity of blood-colored fluid. He was at once relieved of his intense pain. He made a steady recovery, and in about two months had perfect use of the joint.

Case IV.—S. H., aged 38, a carpenter. He had been at work on outside of building most of the winter, and of late had been obliged to kneel on scaffolding a good deal. When I saw him on Feb. 3d, he told me his left knee had been painful and getting stiff for sometime. It was then painful and contained a small amount of fluid. I could not convince him of the importance of giving it rest. I saw him occasionally during the following weeks, during which he was working about and simply using a liniment. On the 11th inst. the condition of the knee being decidedly worse, I told him unless he was willing to give me absolute control of the case, I should do nothing more for him. He was unwilling to submit, and I did not see him again for four days, when he sent for me. The joint was now moderately distended, and the patella floating. I applied posterior splint and elastic bandage, and the patient went to bed. Three days later, the joint being very full, I opened it with a bistoury, letting out a quantity of apparently unchanged synovial fluid. Immediate relief followed, and the bandage was reapplied. The patient was given quinine, iodide of iron, and the kidneys and bowels kept active. But the improvement was not permanent. The joint slowly refilled, and in three weeks it became necessary to evacuate it. This time I used the aspirator, and an accident happened which I am quite sure will not occur to me again. Being ready to introduce the needle, I asked my friend, who was assisting—a most careful and intelligent physician—to exhaust the aspirator bottle. All being ready, I passed the needle into the tissues until the opening near the joint disappeared, when I requested my assistant to turn the stop-cock, and as he did so I thrust the needle into the cavity of the joint. I was startled by a shriek of pain; and I have rarely seen such an agonized expression on any face. The muscles of the leg instantly became rigid and prominent, and the joint increased in size, but no fluid appeared. Fortunately the probable cause of the trouble at once occurred to me. The bottle had not been exhausted but filled with compressed air. The needle was withdrawn, the aspirator made ready and the fluid evacuated as intended. The air which had been driven into the joint bubbled out with the liquid. But the cellular tissue of the thigh contained air which could be felt for two weeks, by which time it had disappeared, leaving no ill effects. Apparently the joint was not injured by its forcible distension, though in four days it had become filled again, and I withdrew with the aspirator fluid of the same appearance as before. In just one week, the other knee having become similarly involved, and the joint cavity very full, I aspirated it. From this time he steadily improved and made a perfect recovery.

I have intentionally omitted to speak of the anatomy of the knee, or of the pathology, diagnosis and classification of the cases I have briefly reported. But it would seem proper to treat them by pressure, absolute rest, and early, and if necessary, repeated evacuations. Barwell, whose reputation in diseases of the joints is certainly second to no man's, says: "If the inflammation be sufficient to cause considerable secretion into the joint, producing marked fullness of the sac, the synovial membrane should be punctured * * * to relieve the tension."

Howard Marsh, in his work on "Diseases of the Joints," says:

"It is now well known that matter, whether connected with acute or chronic arithritis, may be safely evacuated, with the result that the severe suffering, the prolonged fever, the wide and destructive burrowing, and the formation of sinuses, which were the common rule only a few years ago, can be generally avoided."

Dr. G. J. Tobias:

I think the paper is a very good one and the cases reported interesting. The doctor states that in two cases he made an incision before he aspirated; I would ask why he did it, why not try the aspirator first? The accident spoken of has occurred before in this city, that of compressing the air in the bottle instead of pumping it out and producing a vacuum, thereby causing compressed air to be forced into the joint cavity, with some times most serious results. Would it not be a good plan to have some basin or small receptacle at hand containing water, to try the aspirator first and see that it is in proper working order? I wish he would also tell us more fully how he used the posterior splint and whether he padded it, the material used for bandage, etc.

The President:

I take it for granted that Dr. Weston took the ordinary cleanly and antiseptic precautions before incising the knee-joint. Most of us would be inclined to use the aspirator before making an incision, since it is a common observation that after the knee-joint has been aspirated once, twice, three times or more, resolution has occurred and the functions of the joint resumed even when the fluid was purulent. The accident in the use of the aspirator reminds me of one of which I am personally aware as occurring in this city, and which had a more unfortunate termination than in Dr. Weston's case, where most of the air got into the tissue outside of the joint. In the case to which I refer the stop-cock was not turned until the needle had entered the joint, and in three minutes the patient was dead.

Dr. E. B. Weston, in closing the discussion, said:

I used the bistoury instead of the aspirator because it was more convenient at that time. The bistoury has been used a good many times; but I don't pretend to say it is better, perhaps not so good; but in my case it was a matter of convenience. The cases did well. The posterior splint was well padded with cotton. I used for the splint heavy sole leather. An elastic bandage, or rather a flannel bandage, was used below the knee and on the thigh and a rubber bandage to make the pressure over the knee-joint. Of course the aspirator should have been tested. The mistake should not have occurred. And yet no injury was done by the violent distension of the joint, or by the air which entered it.

Dr. William T. Belfield reported

A CASE OF MERCURIAL NECROSIS, WITH SPECIMEN.

The case of mercurial necrosis that I wish to mention is that of a young man, apparently in excellent health, who seven years ago contracted a chancre which was followed by the usual symptoms of constitutional syphilis. He was treated by a regular physician in an eastern city, and for a year and a half had the usual history of a healthy man contracting syphilis — mucous patches, occasional rashes on the skin, etc. After about two and a half years, during which time he was under constant observation and much of the time taking medicine, he had no further indication of constitutional syphilis. It went along that way for about four years. Over six years after the contraction of the chancre, he noticed a slight lump on the instep of the left foot which gave a little pain when he wore tight shoes. Fearing this might be an indication of the return of the disease, he went to his physician who prescribed syphilitic treatment. He ordered the young man to take one-fourth grain of yellow oxide of mercury pill three times a day. The patient, who was naturally anxious to overcome any taint that might remain in him, followed the direction faithfully, and for six months took one of these pills three times a day. At the end of this time his mouth became sore. About ten days after he first noticed the soreness of the mouth, being then in Chicago, he was directed to see me. At that time his gums were quite soft and spongy, his teeth felt loose and he could not bite bread with any comfort. On the left side the alveolar process of the upper jaw was exposed for about three-fourths of an inch in length and one-half inch in vertical breadth. The three molar teeth which were fixed in that portion of the jaw were decidedly loose. The hard palate corresponding to that part of the alveolar process was swollen and there were the usual symptoms of subacute periostitis. He was merely ordered to discontinue the mercury, use a simple mouth wash and take iodide of potassium. In the course of ten days the portion of the jaw exposed was so loose that it could have been removed without difficulty, but the patient, flinching from the slight pain this would cause, declined to have it removed and worked at it with his fingers until, about three weeks after I first saw him, he pulled the piece away. In the meantime he had lost three sound molar teeth. The piece is quite small, merely large enough for the insertion of the three teeth, but it is of some interest. The upper surface of it is the floor of the antrum. During a part of the time the cheek bone was swollen and tender but no abscess formed.

It may be questioned whether this necrosis was mercurial or syphilitic. The man unquestionably, seven years ago, had syphilis, but a little consideration shows that this necrosis was not syphilitic. In the first place, for four and a half years he has had no indication of syphilis; this little hardening of the instep, for which he took the mercury, still persists and is evidently not of syphilitic origin; furthermore, there was thickening and softening of the gums and loosening of the teeth, which was due to the mercury; and, lastly, while syphilis may cause necrosis of the long bones and of many other bones in the body, yet it rarely causes necrosis of the inferior and superior maxillary; while mercury causes necrosis in these bones with especial frequency.

Dr. Belfield said, in reply to a question, that the patient had not been salivated during the time of treatment so far as could be remembered.

Dr. Joseph Haven exhibited

A MECHANICAL SUPPORT FOR MAINTAINING THE LITHOTOMY POSITION.

The position most favorable for the majority of the operations on or about the perineum is that which is known as the lithotomy position. The patient prone upon the back, thighs sharply flexed on the abdomen, knees separated. For the patient to maintain this position unaided, is a difficult procedure, and when under an anæsthetic an absolute impossibility. Hence the necessity for assistance in some form. The usual custom is to entrust each limb to an assistant to hold during the operation. To this procedure there are some objections:

First, it increases the necessary number of assistants by at least two. Usually the patient, if not the whole household, is dreading the operation with nervous anxiety, and the sight of a number of assistants or an unnecessary display of paraphernalia does not tend to quiet their fears or relieve their anxiety, hence operations at the house should be conducted with as little display as possible.

Again, any one who has assisted in holding a patient in the lithotomy position for an hour or so, knows that it is a tiresome undertaking.

Again, emergencies are continually arising where they are called upon to render other assistance, as from their proximity to the operator they are in the way of others, and are called upon to pass instruments, wash sponges, etc., thereby neglecting their *own work* and the falling of the patient's foot in the operator's lap does not tend to *better* the results of the operation or improve the temper of the operator. Consequently it is not strange that various appliances should have been improvised by which the patient could be held in the proper position.

My attention was first called to this subject some six or eight years ago. The operation was for the closing of a lacerated cervix. The operator brought with him an apparatus made of three boards, about four feet long, joined on the sides by hinges. When the side pieces were raised and fastened by hooks the whole formed a sort of trough in which the patient was placed and a harness arrangement bound each limb to the corresponding side piece. While the apparatus worked well upon this occasion, there are still objections to it.

In the first place, it required too long a time to extricate the patient from the apparatus.

Secondly, the effect of putting together such a contrivance in the sick room might be similar to the building of a scaffold in the presence of the condemned.

Thirdly, blood stains are not easily removed from wood, and should there be any septic influence in the case this would be readily absorbed in the pores of the wood; and I doubt the advisability of using a wooden apparatus in repeated operations in close proximity to open wounds.

In a work entitled "Minor Surgical Gynæcology," by Paul Mundé, the author speaks favorably of an apparatus designed by Dr. Hamilton, of Harrisburg, Pa., which he styles "Hamilton's Gynapod." This consists of a piece of wood upon which the buttocks rest; from this arises on either side an arm surmounted by a crutch which supports the knee. While being more simple than the apparatus previously described, some of the same objections hold good in this case. Again, as the limbs are not fastened in any way, it allows too much freedom to the feet. This will be particularly annoying if the operation be a trivial one, not requiring the administration of an anæsthetic.

If then an apparatus for this purpose is of service in these operations, what should be the essential requirements?

First—It should be so constructed that it can be instantly removed if emergency should arise.

Second—It should be of a material that will admit of its being thoroughly cleansed and disinfected.

Third—It should secure the patient firmly and at the same time be as simple, light and easy of transportation as possible.

Acting upon these thoughts, I had made, two years ago, an apparatus based upon the same principle as the one which I shall show you this evening, namely, a light nickel plated bar provided with a hinge in the center, allowing it to be folded up when not in use. This bar passes under the knees of the patient, holding them flexed on the abdomen by means of a band of webbing which passes around the shoulders of the patient; the webbing having a strap and buckle allowing the band to lengthen or shorten according to the size of the patient. Bands of the same material at either end of the rod securely hold the limbs below the knee; every strap being attached to the rod by means of a snap catch, which can be instantly disengaged if occasion so demands. A rod attachment for holding a fountain syringe completes the outfit. The whole apparatus when not in use fits into a case eighteen inches long, five inches deep and four inches wide.

In this connection it becomes my duty, and at the same time a pleasure, to accord to Dr. Thomas B. McBride, of Philadelphia, the credit of having within the last few months devised an apparatus somewhat similar in principle to the one just described. A description of his apparatus will be found in the *Medical and Surgical Reporter* for May. He writes me that he has, since that publication, added the hinge, thereby lessening the length of the apparatus when not in use.

In conclusion, I will add that I have found the apparatus serviceable in all operations where the lithotomy position is indicated, and particularly when operating for slight lacerations of the cervix where no anæsthetic was administered.

The President asked Dr. Haven whether the apparatus answered as well when the patient was under an anæsthetic.

Dr. Joseph Haven:

It answers better. I have used the apparatus a number of times with an anæsthetic and several other physicians have used it. The patient is liable to come out of either before the operation is finished, and with this apparatus they cannot do themselves any harm during the operation.

Dr. Tobias asked Dr. Haven in how many cases of laceration of the cervix he had tried the apparatus, and if the patient ever raised any objection to its use.

Dr. Haven:

Probably twelve or fifteen. No objection has been made to the apparatus except in one case; but you will find that some patients object to any instrument. This patient asked me what it was, and when I explained that it was not an instrument of torture, she readily consented to its use. However, where an anæsthetic is to be given I do not usually put it on until the patient is anæsthetized, and they do not see the instrument at all. It can be put on in less than one minute and taken off in the same time. I very often use it in dressing wounds around the uterus, packing the vagina after opening a pelvic abscess where I do not care to employ an assistant to hold the patient in the lithotomy position, or for slight operations on the rectum,—anything that would not require an anæsthetic.

